# Commentary: Human brain organoid-on-a-chip to model prenatal nicotine exposure

**DOI:** 10.3389/fbioe.2018.00138

**Published:** 2018-10-04

**Authors:** Haowen Qiao, Yu Shrike Zhang, Pu Chen

**Affiliations:** ^1^Department of Biomedical Engineering, School of Basic Medical Sciences, Wuhan University, Wuhan, China; ^2^Hubei Provincial Key Laboratory of Developmentally Originated Disorder, Wuhan, China; ^3^Division of Engineering in Medicine, Department of Medicine, Brigham and Women's Hospital, Harvard Medical School, Cambridge, MA, United States

**Keywords:** brain, Organoid, organ-on-a-chip, prenatal neurological disorder, nicotine exposure

## Brain organoid and brain-on-a-chip technologies

Advances in three-dimensional (3D) neural culture models derived from human stem cells are revolutionizing the paradigm of neuroscience research. These 3D model systems offer a more faithful recapitulation of pivotal functions and cytoarchitectures of *in-vivo* neural tissues than conventional two-dimensional (2D) culture models and animal models (Zhuang et al., 2018).

To establish these 3D neural models, brain organoids (Lee et al., 2017), and brain-on-a-chip systems (Haring et al., 2017) represent two typical approaches based on the strategies of developmental biology and bioengineering, respectively. Brain organoids are formed by sequential steps in neural development *in vitro*, including generation of embryoid bodies (EBs) from stem cells, induction of neuroectoderm in a dish, expansion of neuroepithelium in Matrigel, and organoid differentiation in suspension (Jo et al., 2016). Brain organoids explore the power of developmental biology to reproduce the early stages of fetal brain development, including polarized neuroepithelium, cell type heterogeneity and segregation of discrete brain regions (Luo et al., 2016). In contrast, brain-on-a-chip systems provide better capacity to reconstitute *in-vivo* neural microenvironments including intercellular interactions, extracellular matrix (ECM), and hemodynamics, in a deterministic manner. However, both of these approaches have their own limitations. For instance, the brain organoid culture systems usually have very limited controllability over biochemical and biophysical factors in the surrounding 3D microenvironment, while the brain-on-a-chip systems are unable to reconstitute the biological complexity existing during the brain development.

By combining the advantages of both organ-on-a-chip and organoid technologies through a synergistic strategy, the organoid-on-a-chip platform has emerged as a new model to recapitulate the essential structural and physiological features of the *in vivo* tissue and the corresponding 3D tissue microenvironment (Skardal et al., 2016; Takebe et al., 2017). Following this concept, a recent paper from the Qin Group (Wang et al., 2018) demonstrated a brain organoid-on-a-chip system, which displayed remarkable capacity for a better study of prenatal neurological disorders.

## Brain organoid-on-a-chip

Nicotine is recognized as a neurotoxin to trigger various neural dysfunctions and long-lasting deficit when exposing to the fetal brain (Pauly and Slotkin, 2008). Moreover, neurobehavioural disorders associated with maternal smoking during pregnancy are related to offspring intelligence (Breslau et al., 2005), anxiety behaviors (Moylan et al., 2015), cognitive ability (Batty et al., 2006), and neurodegenerative diseases of adulthood (Picciotto, 2008). However, the understanding of human neurological disorder under prenatal nicotine exposure (PNE) is an outstanding challenge due to the differences in histomorphology, physiology, and spatiotemporal patterns of neurodevelopment between humans and mice. The study by the Qin Group (Wang et al., 2018) established a brain organoid-on-a-chip system to model neurodevelopmental disorders for investigating PNE *in vitro*.

**Figure 1:**
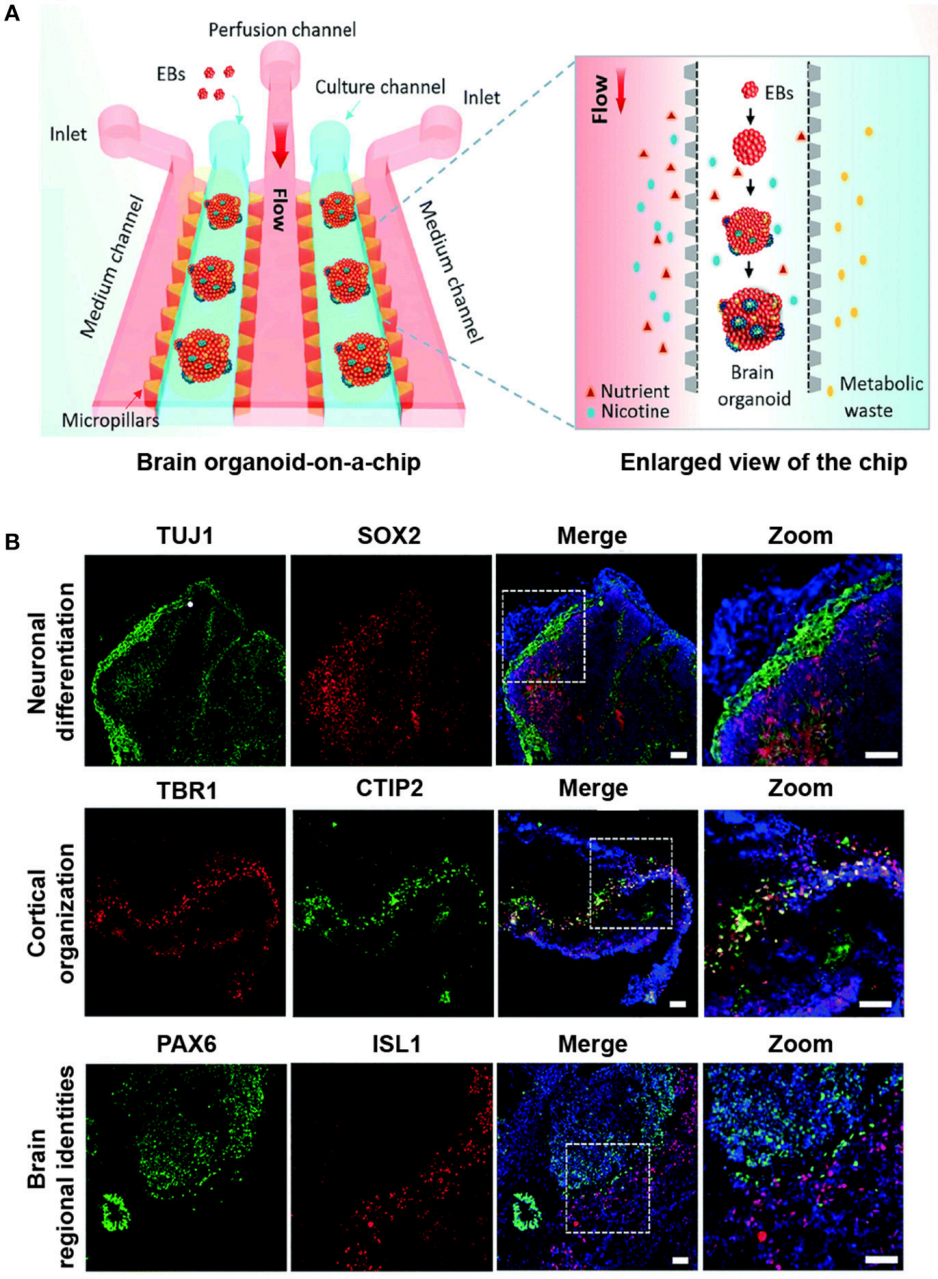
Modeling prenatal nicotine exposure employing human brain organoid-on-a-chip. **(A)** The schematic diagram of the brain organoid-on-a-chip system for modeling prenatal nicotine exposure. The brain organoid-on-a-chip system was established by sequential steps including EBs culture, neural differentiation and formation of brain organoids by integrating 3D Matrigel and fluid flow. By using this system, the effects of nicotine exposure on brain development at early stages were explored at early stages. **(B)** Immunohistochemical staining was performed for the expression of neural progenitor marker SOX2(red), neuronal marker TUJ1 (green), cortical layer markers TBR1(red) and CTIP2 (green), specific forebrain marker PAX6 (green), and hindbrain marker ISL1(red) in brain organoids on day 33. Nuclei are stained with DAPI (blue). Scale bars = 50 μm. Adapted from Wang et al. (2018). (http://dx.doi.org/10.1039/C7LC01084B) with permission of The Royal Society of Chemistry.

The brain organoid-on-a-chip consisted of two culture channels, one perfusion channel and two medium channels (Figure 1A). To generate the brain organoid, EBs mixed in Matrigel were pipetted into each culture channels for *in situ* 3D differentiation. The differentiation microenvironment on the chip recapitulated the *in vivo* fetal brain development by precisely controlling the biochemical and mechanical cues.

Consistent with the early stages of human brain organogenesis, this synergistically engineered model clearly revealed the feasibility of well-recognized neuronal differentiation, diverse brain regional identities, and cortical organization in the microfluidic chip (Figure 1B). In the areas mimicking neural differentiation, the organoids showed a high proportion of distinct neural identities, including markers for neural progenitor cells (SOX2) and neurons (TUJ1). Likewise, the different brain regional identities during brain organoid development were stained positive by the specific markers such as PAX6 and ISL1, suggesting the early developing forebrain and hindbrain. Furthermore, the layered cortical architecture could verify the initiation of the cortical plate layer formation, which was characterized by the deep-layer marker CTIP2 located adjacent and internal to the TBR1-positive pre-plate.

To investigate neurodevelopment under PNE, the authors focused on the effect of varying doses of nicotine on the development of brain organoids. During brain organogenesis on the chip, nicotine treatment led to premature neuronal differentiation and disruption of brain regionalization. Moreover, the authors utilized the brain organoids-on-a-chip model to examine nicotine-induced impaired cortical development from days 35 to 40, and the data might contribute to a better understanding of various postnatal cognitive dysfunctions under PNE. Intriguingly, these brain organoids exposed to nicotine showed the abnormal neurite outgrowth at a dose-dependent manner. The similar brain organoid-on-a-chip system has also been used to reveal the mechanism of the impaired neurogenesis under exposure to cadmium (Yin et al., 2018) and alcohol (Zhu et al., 2017).

Compared to the previous 3D neural culture models (Lancaster et al., 2013; Bouyer et al., 2016) which were based solely on the engineering approach or the developmental biology approach, the bioengineered brain organoids used in this study (Wang et al., 2018) more truthfully mimicked the brain development under PNE by integrating brain organoid in a microfluidic device. In addition, optically transparent property of the microfluidic device facilitated *in situ* real-time imaging of the neurodevelopmental process and the brain organoid responses to nicotine.

## Future perspective

A clear understanding of the early stages of human embryonic development is essential for a thorough investigation into the effects of PNE on human brain development. Despite that great efforts have been devoted to interrogating the effects of PNE on rodent models (Abreu-Villaça et al., 2004), the understanding of the human brain development under PNE remains elusive due to the significant difference between human and rodent physiology. Brain organoid-on-a-chip system utilized in the current work (Wang et al., 2018) could possibly serve as an alternative human-relevant neurodevelopmental model, eliminating ethical concerns regarding human clinical trials in smoking pregnant women.

For basic and translational neuroscience research, generation of standardized and homogenous brain organoid is critical but remain an unsolved issue in this system. Nevertheless, brain organoid-on-a-chip system opens a new avenue for modeling the human prenatal neurodevelopmental disorders. We expect that this commentary will stimulate motivation to expand the applications of organoid-on-a-chip systems and more complex multi-organoid-on-a-chip systems in screening of drug candidates, probing of disease mechanisms, as well as advancing novel therapies.

## Author contributions

YZ and PC conceived the idea. HQ, YZ, and PC wrote the paper.

### Conflict of interest statement

The authors declare that the research was conducted in the absence of any commercial or financial relationships that could be construed as a potential conflict of interest.
